# Predicting clinically significant prostate cancer using DCE-MRI habitat descriptors

**DOI:** 10.18632/oncotarget.26437

**Published:** 2018-12-14

**Authors:** Andres N. Parra, Hong Lu, Qian Li, Radka Stoyanova, Alan Pollack, Sanoj Punnen, Jung Choi, Mahmoud Abdalah, Christopher Lopez, Kenneth Gage, Jong Y. Park, Yamoah Kosj, Julio M. Pow-Sang, Robert J. Gillies, Yoganand Balagurunathan

**Affiliations:** ^1^ Department of Cancer Physiology, H.L. Moffitt Cancer Center, Tampa, FL, USA; ^2^ Department of Radiology, Tianjin Medical University Cancer Institute and Hospital, Tianjin, China; ^3^ Department of Radiation Oncology, University of Miami Miller School of Medicine, Miami, FL, USA; ^4^ Department of Urology, University of Miami Miller School of Medicine, Miami, FL, USA; ^5^ Department of Radiology, H.L. Moffitt Cancer Center, Tampa, FL, USA; ^6^ Department of Cancer Epidemiology, H.L. Moffitt Cancer Center, Tampa, FL, USA; ^7^ Department of Radiation Oncology, H.L. Moffitt Cancer Center, Tampa, FL, USA; ^8^ Department of Urology, H.L. Moffitt Cancer Center, Tampa, FL, USA

**Keywords:** radiomics of MRI, DCE, mpMRI, prostate, prostate imaging

## Abstract

Prostate cancer diagnosis and treatment continues to be a major public health challenge. The heterogeneity of the disease is one of the major factors leading to imprecise diagnosis and suboptimal disease management. The improved resolution of functional multi-parametric magnetic resonance imaging (mpMRI) has shown promise to improve detection and characterization of the disease. Regions that subdivide the tumor based on Dynamic Contrast Enhancement (DCE) of mpMRI are referred to as *DCE-Habitats* in this study. The DCE defined perfusion curve patterns on the identified tumor habitat region are used to assess clinical significance. These perfusion curves were systematically quantified using seven features in association with the patient biopsy outcome and classifier models were built to find the best discriminating characteristics between clinically significant and insignificant prostate lesions defined by Gleason score (GS). Multivariable analysis was performed independently on one institution and validated on the other, using a multi-parametric feature model, based on DCE characteristics and ADC features. The models had an intra institution Area under the Receiver Operating Characteristic (AUC) of 0.82. Trained on Institution I and validated on the cohort from Institution II, the AUC was also 0.82 (sensitivity 0.68, specificity 0.95).

## INTRODUCTION

Prostate cancer is the second largest cause for cancer deaths among men in the US with an estimated 21% of newly diagnosed cancers [1]. Over-diagnosis and resulting overtreatment of the disease is a major concern, some of which is attributed to the traditional screening procedures, including prostate specific antigen (PSA) [2–4]. mpMRI has improved the detection of clinically significant lesions [5] impacting the staging, diagnosis and follow-up of patients with prostate cancer [6]. Dynamic Contrast Enhancement (DCE) imaging is routinely included in prostate MRI exams along with T2-weighted imaging (T2W), and diffusion-weighted imaging (DWI). DCE imaging shows the dynamics of the administered contrast agent, while Apparent Diffusion Coefficient (ADC) qualifies tissue density by measuring diffusion of water molecules. Clinical assessment of lesions on MRI is guided by the Prostate Imaging Reporting and Data System, version 2 (PIRADSv2) standard [7]. The standard reporting restricts use of DCE to peripheral zone (PZ) when clinical significance of the lesion in DWI is equivocal (DWI PIRADSv2 score of three). DCE analysis can be broadly divided in two approaches: Quantitative, where a model is used to determine the rate of contrast transfer from blood into tissue, and semi-quantitative, where the analysis uses time activity curve characteristics to describe different contrast absorption patterns. The difficulty in establishing consistent features from the DCE curves, as well as the high inter-observer variability affects the use of DCE in a quantitative fashion. Even though semi-quantitative DCE analysis lacks a formal modeling of contrast uptake, it provides a more general characterization of the activity curves. This characterization has been used in the automatic detection and quantitative scoring of prostate cancer aggressiveness [8–10]. Quantitative models study the pharmacokinetics of the contrast agent in the prostate, especially with the use of arterial input function (AIF) [11]. The model proposed by Tofts et al [12] provides an elegant model to characterize contrast absorption. It has been shown that the pharmacokinetic analysis parameters correlate with lesion aggressiveness but with high inter-institution variability [13]. Major constraint in the identification of a region to derive a reference AIF has prevented these features from being used in practice [14].

In this study, we examine the utility of DCE derived quantitative characteristics on a habitat region co-localized by ADC to discriminate clinically significant prostate cancer. We also show ways to improve discriminatory ability by adding radiomics features derived on an ADC region. The identified model was validated in an independent cohort obtained from a different institution.

## RESULTS

Data set from ***Institution I*** consisted of 173 positive for cancer biopsies from 57 patients; data set from ***Institution II*** consisted of 51 biopsies from 39 patients. Biopsies without assigned Gleason Score (GS) were discarded, such as those labeled by the clinical pathologist as benign prostatic tissue, or as benign prostatic hyperplasia. Biopsies with an assigned GS sum of 6 or above were included for analysis. The average interval between imaging and biopsy sampling was 12 days for ***Institution I*** and 27 days for ***Institution II***. The data from ***Institution I*** consisted of 116 clinically insignificant and 57 clinically significant biopsies. The data from ***Institution II*** consisted of 22 clinically insignificant and 29 clinically significant biopsies. Patients with temporal resolution larger than or equal to 15 sec were excluded (***Institution I***, n=14; ***Institution II***, n=6). Patients with DCE motion artifacts were also excluded (***Institution I***, n=5; ***Institution II***, n=3). The final analysis included 38 patients (99 biopsies; 84 clinically insignificant, 15 clinically significant: nine with GS 3+4, four with GS 4+3, and two with GS 5+3) for ***Institution I*** and 30 patients (42 biopsies; 17 clinically insignificant, 25 clinically significant: sixteen with GS 3+4, six with GS 4+3, two with GS 4+4 and one GS 4+5) for ***Institution II***. Prior preliminary study had shown detrimental effects of using low temporal resolution on the estimated curve characteristics [15]. The intra-modality temporal alignment of DCE was measured as the percentage difference between the mean prostate time activity curve and its fitted model. Before registration, the mean difference was 11.17% (standard deviation, 7.64%). After registration the mean difference was 7.77% (standard deviation, 2.58%).

In this study, classifier models using features on the perfusion characteristics were used to discriminate between clinically insignificant and significant prostate cancer (see Table 1). The highest predictive DCE and ADC features were used to develop a multivariable predictor model. The *wash-in slope* habitat and the radiologist contours had a Dice score of 0.21 suggesting that this habitat was exploring the peritumoral region, adding information from the surrounding environment to the model. Intra-institution analysis of DCE features (diagonal, Table 2) showed that the AUC for ***Institution I*** was in the range 0.58 to 0.70 and for ***Institution II*** it was in the range 0.37 to 0.71. For both institutions, the top feature based predictors were *slope product* and *finalAUC*. Pairwise analysis of DCE features (off diagonal, Table 2) showed that for ***Institution I***, the AUC increased for the pair of features (*wash-in slope*, *initialAUC*) to 0.77, with sensitivity of 0.68 and specificity of 0.85. For ***Institution II***, the AUC increased for the pair of features (*time-to-peak*, *final AUC*) to 0.82, with sensitivity of 0.84 and specificity of 0.79.

**Table 1:** List of DCE features analyzed in this paper

#	Feature ID	Feature Description
1	*s_p_*	peak enhancement, *s_m_*-*s_0_*
2	*tau*	time-to-peak
3	*wi*	wash-in slope
4	*wo*	wash-out slope
5	*AUCi*	initial AUC, AUC_t0-t0+60_
6	*AUCf*	final AUC, AUC_t0+240-t0+270_
7	*m_io_*	slope product, wi^*^wo

Each DCE feature generates a 3D map that is thresholded to converge to a 3D DCE volume. For each feature, it is shown the 2D Dice coefficient between each converged volume and the manual radiologist contour of the finding, for the slice with the largest manual volume.

**Table 2:** Intra-institution evaluation of pairs of DCE features

Institution I								Institution II							
**Sensit.**	***s_p_***	***tau***	***wi***	***wo***	***AUCi***	***AUCf***	***m_io_***	**Sensit.**	***s_p_***	***tau***	***wi***	***wo***	***AUCi***	***AUCf***	***m_io_***
*s_p_*	0.59	0.63	0.66	0.64	0.66	0.67	0.71	*s_p_*	0.58	0.37	0.47	0.63	0.53	0.53	0.47
*tau*		0.63	0.78	0.71	0.66	0.74	0.67	*tau*		0.37	0.53	0.53	0.47	0.84	0.74
*wi*			0.58	0.68	0.68	0.68	0.71	*wi*			0.53	0.58	0.58	0.53	0.79
*wo*				0.51	0.59	0.71	0.60	*wo*				0.47	0.63	0.74	0.63
*AUCi*					0.63	0.73	0.71	*AUCi*					0.68	0.74	0.53
*AUCf*						0.68	0.75	*AUCf*						0.79	0.58
*m_io_*							0.67	*m_io_*							0.63
**Specif.**	*s_p_*	*tau*	*wi*	*wo*	*AUCi*	*AUCf*	*m_io_*	**Specif.**	*s_p_*	*tau*	*wi*	*wo*	*AUCi*	*AUCf*	*m_io_*
*s_p_*	0.56	0.75	0.60	0.74	0.60	0.77	0.67	*s_p_*	0.42	0.42	0.53	0.58	0.42	0.53	0.47
*tau*		0.64	0.67	0.68	0.82	0.73	0.70	*tau*		0.37	0.37	0.58	0.42	0.79	0.68
*wi*			0.60	0.67	0.85	0.68	0.70	*wi*			0.37	0.63	0.37	0.63	0.74
*wo*				0.67	0.68	0.73	0.73	*wo*				0.74	0.58	0.63	0.58
*AUCi*					0.63	0.77	0.74	*AUCi*					0.58	0.53	0.37
*AUCf*						0.70	0.75	*AUCf*						0.63	0.58
*m_io_*							0.73	*m_io_*							0.79
**AUC**	*s_p_*	*tau*	*wi*	*wo*	*AUCi*	*AUCf*	*m_io_*	**AUC**	*s_p_*	*tau*	*wi*	*wo*	*AUCi*	*AUCf*	*m_io_*
*s_p_*	0.58	0.69	0.63	0.69	0.63	0.72	0.69	*s_p_*	0.50	0.39	0.50	0.61	0.47	0.53	0.47
*tau*		0.64	0.73	0.70	0.74	0.73	0.68	*tau*		0.37	0.45	0.55	0.45	0.82	0.71
*wi*			0.59	0.68	0.77	0.68	0.71	*wi*			0.45	0.61	0.47	0.58	0.76
*wo*				0.59	0.64	0.72	0.66	*wo*				0.61	0.61	0.68	0.61
*AUCi*					0.63	0.75	0.73	*AUCi*					0.63	0.63	0.45
*AUCf*						0.69	0.75	*AUCf*						0.71	0.58
*m_io_*							0.70	*m_io_*							0.71

Sensitivity, specificity and AUC for classification between clinically insignificant and significant cancer is shown, based on MRI-guided biopsies. Decision trees were used as classifiers. Leave-one-out(LOO) cross validation was used. The diagonal corresponds to the univariate case.

Statistical analysis showed that for **Institution I**, the feature with the largest number of naïve pair-wise significantly different AUC (Supplementary Table 1, **Feature 27**) was the pair (*final AUC*, *slope product)*. After correcting for multiple comparisons, the significance level was adjusted to 0.0137 and only 11 out of 27 experiments had significantly different AUC (Table 3). For **Institution II**, the best pair (Supplementary Table 2, **Feature 12**) was the pair (*time-to-peak*, *final AUC*). The significance level was corrected to 0.0321, resulting in 22 out of 27 significantly different AUC curves. The remaining 5 feature tuples included either *final AUC* or *slope product*. Additionally, they correspond to the top-performing AUC in Table 2, outlining a cluster of well-performing features (Table 4). The same intra-institution analysis of DCE features was performed for ***Institution II*** without image registration (diagonal, Table 5). It showed that the AUC was in the range 0.44 to 0.59, with the top predictor being *initialAUC*. Pairwise analysis of DCE features (off diagonal, Table 5) showed that for ***Institution II***, the AUC increased for the pair of features (*time-to-peak*, *initialAUC*) to 0.73, with sensitivity of 0.84 and specificity of 0.63. ADC features were ranked by the intra institution AUC and the top five pairs (Table 6, description of features in Table 7) were considered for inter-institution analysis. AUC for both ***Institution I*** and for ***Institution II*** were in the range 0.71 to 0.82. The top performing ADC features were associated with histogram gradient, volume/intensity fraction difference and habitat volume.

**Table 3:** Significant differences in AUC for Institution I

Institution I							
**pValue**	***s_p_***	***tau***	***Wi***	***wo***	***AUCi***	***AUCf***	***m_io_***
*s_p_*	0.054	0.307	0.008	0.064	0.008	0.012	0.076
*Tau*		0.000	0.001	0.053	0.058	0.026	0.008
*Wi*			0.000	0.035	0.009	0.035	0.054
*Wo*				0.037	0.008	0.575	0.027
*AUCi*					0.022	0.337	0.182
*AUCf*						0.014	1.000
*m_io_*							0.006

DeLong test was used between the DCE feature tuple (*AUCf, m_io_*) and all other tuples to establish statistical difference. Significance level (α) was set to 0.05. False discovery rate (FDR) was used to correct for multiple comparisons, with an adjusted α (*adj_*α) = 0.0137.

**Table 4:** Significant differences in AUC for Institution II

Institution II							
**pValue**	***s_p_***	***tau***	***wi***	***wo***	***AUCi***	***AUCf***	***m_io_***
*s_p_*	0.005	0.032	0.001	0.017	0.010	0.002	0.014
*Tau*		0.032	0.013	0.007	0.009	1.000	0.258
*Wi*			0.013	0.031	0.008	0.022	0.471
*Wo*				0.024	0.017	0.084	0.017
*AUCi*					0.021	0.009	0.013
*AUCf*						0.369	0.027
*m_io_*							0.290

DeLong test was used between the DCE feature tuple (*tau*, *AUCf*) and all other tuples to establish statistical difference. Significance level (α) was set to 0.05. False discovery rate (FDR) was used to correct for multiple comparisons, with an adjusted α (*adj_*α) = 0.0321.

**Table 5:** Evaluation of pairs of DCE features for Institution II, without image registration

Institution II							
**Sensit.**	*s_p_*	*tau*	*wi*	*wo*	*AUCi*	*AUCf*	*m_io_*
*s_p_*	0.80	0.64	0.84	0.64	0.72	0.52	0.68
*tau*		0.56	0.56	0.52	0.84	0.72	0.80
*wi*			0.60	0.76	0.72	0.68	0.68
*wo*				0.60	0.88	0.76	0.68
*AUCi*					0.84	0.68	0.68
*AUCf*						0.84	0.44
*m_io_*							0.44
**Specif.**	*s_p_*	*tau*	*wi*	*wo*	*AUCi*	*AUCf*	*m_io_*
*s_p_*	0.38	0.31	0.44	0.44	0.69	0.19	0.44
*tau*		0.31	0.38	0.44	0.63	0.19	0.50
*wi*			0.38	0.38	0.75	0.31	0.38
*wo*				0.31	0.31	0.50	0.44
*AUCi*					0.44	0.31	0.44
*AUCf*						0.25	0.19
*m_io_*							0.44
**AUC**	*s_p_*	*tau*	*wi*	*wo*	*AUCi*	*AUCf*	*m_io_*
*s_p_*	0.59	0.48	0.64	0.54	0.70	0.35	0.56
*tau*		0.44	0.47	0.48	0.73	0.45	0.65
*wi*			0.49	0.57	0.74	0.50	0.53
*wo*				0.46	0.60	0.63	0.56
*AUCi*					0.64	0.50	0.56
*AUCf*						0.55	0.31
*m_io_*							0.44

Sensitivity, specificity and AUC for classification between clinically insignificant and significant cancer is shown, based on MRI-guided biopsies. Decision trees were used as classifiers. Leave-one-out(LOO) cross validation was used. The diagonal corresponds to the univariate case.

**Table 6:** Intra-institution evaluation pair-wise, bivariate/variable ADC features

ADC Features		Institution I, LOO			Institution II, LOO		
		Sensitivity	Specificity	AUC	Sensitivity	Specificity	AUC
MaxHistGrad	MinorAxisL	0.79	0.84	0.82	0.74	0.79	0.76
MaxHistGrad	SurfArea	0.82	0.77	0.79	0.63	0.84	0.74
MaxHistGrad	MinHistGrad	0.75	0.67	0.71	0.74	0.89	0.82
VolIFractDiff	LeastAxisL	0.68	0.73	0.71	0.74	0.89	0.82
IntVFractDiff	LeastAxisL	0.84	0.75	0.79	0.58	0.84	0.71

Pairings of 90 ADC features for intensity statistics, histogram and shape were used for classification between clinically insignificant and significant cancer. Sensitivity, specificity and AUC were computed. The cumulative AUC between institutions was used for ranking. The top five pairings are shown below. Decision trees were used as classifiers. Leave-one-out (LOO) cross validation was used for intra-institution evaluation

**Table 7:** Description of top performing ADC features

#	Feature ID	Feature Description
1	MaxHistGrad	Maximum Histogram Gradient Grey Level
2	MinHistGrad	Minimum Histogram Gradient Grey Level
3	VolIFractDiff	Volume at Intensity Fraction Difference
4	IntVFractDiff	Intensity at Volume Fraction Difference
5	SurfArea	Surface Area (mm^2^)
6	MinorAxisL	Minor Axis Length
7	LeastAxisL	Least Axis Length

Multivariable analysis was performed by joining pairs of DCE features (Table 2) with the top five performing ADC pairs (from Table 6) as predictors and evaluating their predictive power. The quadruples were ranked by the cumulative inter institution AUC. The top performing couples corresponded to the same ADC feature pair: (*MaxHistGrad*, *MinorAxisL*). For intra-institution analysis (Table 8) the AUC for ***Institution I*** was in the range 0.75 to 0.88, and for ***Institution II*** it was in the range 0.45 to 0.76. For inter-institution analysis (Table 9) the AUC for ***Institution I*** was in the range 0.71 to 0.82, and for ***Institution II*** it was in the range 0.54 to 0.70.

**Table 8:** Intra-institution evaluation of pair-wise DCE and ADC features

DCE Features + 2 ADC Features (MaxHistGrad, MinorAxisL)		Institution I, LOO			Institution II, LOO		
		Sensitivity	Specificity	AUC	Sensitivity	Specificity	AUC
*Tau*	*AUCf*	0.84	0.70	0.77	0.58	0.42	0.50
*Wi*	*wo*	0.78	0.73	0.75	0.58	0.42	0.50
*AUCi*	*m_io_*	0.89	0.86	0.88	0.68	0.84	0.76
*AUCi*	*wo*	0.88	0.74	0.81	0.68	0.74	0.71
*s_p_*	*m_io_*	0.90	0.79	0.85	0.47	0.42	0.45

Pairings of 7 DCE features (Table 1) combined with the top performing 5 ADC (Table 3). Sensitivity, specificity and AUC were computed. The cumulative inter-institution AUC was used for ranking. Leave-one-out (LOO) cross validation was used for decision tree classifiers. All top DCE-ADC tuples had the same ADC pair (MaxHistGrad, MinorAxisL)

**Table 9:** Inter-institution evaluation of pair-wise DCE and ADC features

DCE Features + 2 ADC Features (MaxHistGrad, MinorAxisL)		Institution I → Institution II			Institution II → Institution I		
		Sensitivity	Specificity	AUC	Sensitivity	Specificity	AUC
*Tau*	*AUCf*	0.58	0.95	0.76	0.67	0.73	0.70
*Wi*	*wo*	0.53	0.89	0.71	0.67	0.73	0.70
*AUCi*	*m_io_*	0.47	0.95	0.71	0.78	0.58	0.68
*AUCi*	*wo*	0.68	0.95	0.82	0.38	0.70	0.54
*s_p_*	*m_io_*	0.63	0.95	0.79	0.38	0.74	0.56

The top performing tuples in the intra-institution DCE and ADC feature evaluation (Table 5) are independently tested between institutions. Sensitivity, specificity and AUC were computed. Decision trees were used as classifiers.

## DISCUSSION

DCE features show promise in discriminating between normal appearing versus tumor tissue: In a recent study [9], characterization of the prostate region (radiomics) in MRI showed predictive of cancer tissue, with an AUC of 0.71 for PZ and of 0.68 for TZ. DCE features have also shown to be discriminant between clinically significant and insignificant prostate cancer: *wash-in* and *wash-out slope* were two of the parameters in a three-variable linear models that showed a classification AUC of 0.85 for PZ and 0.92 for TZ in an intra-institution setting using whole-mount histopathology contours registered unto T2W for lesion characterization [10]. The discriminatory power of DCE shows promise in this work; only if the procedure could be translated in clinical practice to obtain better risk stratification therefore avoiding aggressive treatment in patients with non-significant cancer. It was already shown that DCE-based habitats provide significant correlation between clinically insignificant and significant lesions in [8] where the AUC for the significant quantitative features reached 0.88 and 0.95. This previous work supports the underlying hypothesis for this study: that DCE features are able to differentiate clinical significance of identified lesions. It is shown in this paper that DCE and ADC radiomics features from a *wash-in slope* induced habitat differentiate clinically significant vs insignificant cancer with an AUC of 0.88 and 0.82 for intra and inter-institution analysis respectively (Tables 5 and 6) showing similar discriminating power than whole-mount histopathology-based regions of interest [10].

The intent of this paper is to show utility of DCE habitats accurate predict cancer status and to show adding multiple modality information (ADC metrics) shown improvement in the predictability. The analysis presented here did not break down the tumors by prostatic zone because of the small sample size for clinically significant lesions, but the segmentation step was aware of the prostate zone containing the largest percentage of the lesion.

Although DCE plays a minimal role in PIRADSv2, finer quantification of perfusion characteristics may have a greater role. As shown in this study, pairs of DCE features had an AUC of 0.71 for ***Institution I***, and 0.82 for ***Institution II***, for intra-institution analysis. For inter-institution analysis (Table 6), it can be seen that training on ***Institution I*** had better performance than training on ***Institution II***. This might be due to the difference in training size (99 and 42 biopsies, respectively) suggesting than the radiomics approach requires a larger training set. The best performing features in this inter-institution analysis (Table 6) for training on ***Institution I*** were the tuple (*AUCi*, *wo*, *MaxHistGrad*, *MinorAxisL*) with an AUC of 0.82. This tuple had both features from early uptake (*AUCi*) and late uptake (*wo*) suggesting that including descriptors for the whole DCE curve improves performance. The ADC features suggest that abrupt intensity changes and the volume of the ADC habitat play an important role in improving classification. Perfusion characteristics associated with early enhancement (peak enhancement, time-to-peak, start of enhancement) continued to be some of the top predictors of clinical significance. In addition, it was found that the rate of contrast activity at early and late absorption (wash-in slope, wash-out slope, and slope product) were consistently top candidates related to Gleason tumor grades. A meta-analysis of various DCE publications in prostate cancer [16] showed that the forward volume transfer (Ktrans) and reverse reflux (Kep) are consistently related to tumor aggressiveness and these measures were valuable for differential diagnosis of prostate cancer. This study found that feature descriptors related to perfusion peak, rate and wash-out characteristics were predictive of clinical significance. Multicenter validation studies in breast cancer finds variation in concordance between participants estimate of Ktrans, ranging from 0.047 to 0.92 [17].

There are a few studies using quantitative imaging in prostate cancer relating features to aggressiveness. Some top features correspond to gradients, Gabor filters, etc [18]. The predictors showed high specificity (>95%) but a low level of sensitivity (≤ 42%). In a recent review on prostate cancer, the concern of over-diagnosis was addressed by a suggestion to exploit quantitative imaging metrics to offset the need for invasive biopsies [19]. A quantitative imaging approach such as the one presented in this current study has the potential to significantly reduce the number of biopsies and associated morbidity. The presented approach of using a sphere around the lesion to find an appropriate habitat can easily be adapted to a deep-learning framework to identify DCE habitats in a data-driven fashion that shows promising in imaging but requires larger data sets. Center of mass of manually drawn contour was used, to co-localized ADC map to converge on DCE habitats. Small changes in lesion contours will have minimal impact on the habitat region.

The need for registration between different modalities of medical imaging has been well documented [20]. The measurement of registration accuracy is still challenging for 4D DCE data. DCE intensity variances over time have been used as a similarity measure in registration of DCE data [21]. We used MIM PACS registration modules (FDA approved package) accessed iteratively using custom routines to minimize discrepancy in mpMRI modality alignment. Based on our preliminary analysis to study the influence of modality alignment to downstream analysis, we find *time-to-peak* and *initialAUC* are early enhancement features, that are probably not affected by patient movement which predominantly happens during the later parts of the scan. The aim of the work presented here is not to identify nor delineate suspicious regions in the prostate. Our goal is to provide the radiologists and oncologists with an accurate prediction of the clinical significance of identified lesions.

The American College of Radiology recommends use of high DCE temporal resolution (10 seconds or less) for characterizing prostatic vasculature [22]. In a recent study, a sampling resolution of 15 sec and above resulted in a statistical insignificance compared to higher resolutions [15]. Due to retrospective nature of the study, data sets with temporal sampling larger than 15 seconds were removed to compromise on the sample size between two centers.

## MATERIALS AND METHODS

### Patient data

This study evaluated the performance of features using two independent data sets: First cohort was collected at the University of Miami (***Institution I***) under approved Institutional Review Board (IRB) protocol and de-identified for retrospective analysis. An additional cohort was collected at H. Lee Moffitt Cancer Center (***Institution II***), under protocol approved by the University of South Florida's IRB. The patient's informed consent was waived for retrospective access of de-identified patient records. The methods were performed in accordance with the approved guidelines. Data consisted of histopathology analysis of prostate biopsies acquired with either template or targeted biopsy from pre-treatment MRI acquired by fusing the MRI and real-time ultrasound images using Uronav (Invivo Corporation, Gainesville, FL), which allows for accurate measurement of needle location. For this study, GS values were grouped in two categories: *clinically insignificant cancer* (=GS6) and *clinically significant cancer* (GS ≥ 7). All statistics were performed using this grouping.

### MRI acquisition and pre-processing

Routine clinical mpMRI acquisition includes T2W, DCE, and diffusion weighted imaging (DWI). DWI includes an ADC map generated at acquisition time. ***Institution I*** imaging was acquired using multiple scanners, Siemens (Siemens, Munich, Germany) and GE (General Electrics, Boston, MA) with 19 and 38 patients, respectively. Both acquired at 3T with an external pelvic coil. DWI was acquired using three b-values: 50, 500 and 1000 (n=37) and 50, 500 and 1400 (n=20). For DWI, the median repetition time (TR) was 9.5 sec (range 6.6-9.87 sec) and the median echo time (TE) was 55.8 msec (range 52.4-93 msec). For DCE, the median TR was 4.05 msec (range 3.04-5.24 msec), the median TE was 1.78 msec (range 1.36-2.33 msec), flip angle was 12 deg (n=54) and 10 deg (n=3), temporal resolution was 7 sec (n=43) and 30 sec (n=14). ***Institution II*** imaging was also acquired using multiple scanners, Siemens (Siemens, Munich, Germany), Philips (Philips, Amsterdam, Netherlands), and GE (General Electrics, Boston, MA) with 31, 5, and 3 patients, respectively. Acquired using 3T (n=7) and 1.5T (n=32) with an endorectal coil (eCoil, Medrad, Pittsburgh, PA). For DWI, the median TR was 7.4 sec (range 3.2-9.5 sec) and the median TE was 95 msec (range 70.5-115 msec). For DCE, the median TR was 4.72 msec (range 2.42-4.72 msec), the median TE was 1.34 msec (range 1.06-2.08 msec), flip angle was 12 deg (n=34) and 10 deg (n=5), temporal resolution was 11 sec (n=33) and 16.5 sec (n=6).

All modalities were registered locally to the prostate using the T2W image as reference. We used gradient descent of mutual information on the space spanned by 3D affine transformations, using a combination of native and custom routines on the MIM PACS software (MIM Corporation, Cleveland, OH). Manual contours of the prostate, PZ, and the radiologist finding in the pre-biopsy MRI were stored as RT-DICOM structures. The peak-absorption time point S_peak_ was identified in DCE using the AIF signal as reference. All other DCE time points were registered to S_peak_. ADC were standardized within the prostate, i.e., ADCz = (ADC-mean(ADC(prostate)))/std(ADC(prostate)), which has been shown to be used to standardize data variability [23]. DCE data was normalized using an automatically segmented arterial contour as described in [24], which makes the signal proportional to the change in relaxation rate caused by the contrast agent weighted by the initial spin-lattice relaxation time [25]. Image analysis was performed using custom routines written in Matlab (Mathworks, Natick, MA) which were accessed directly from the PACS (MIM Corporation, Cleveland, OH, USA).

### Image registration in mpMRI

In order to minimize the effects of patient movement during the long period of mpMRI scan on the downstream analysis has motivated to use image registration to align modalities [20]. The measurement of registration accuracy is still challenging for 4D DCE data. In prior studies intensity variances over time have been used as a similarity measure in registration of DCE data [21]. This variance was quantified by measuring the percentage difference between the mean prostate time activity curve and its fitted model. The mean percentage different of the signal intensity decreased from 11.17% without any to 7.77% after image registration. The standard deviation also reduced, from 7.64% to 2.58%, showing a larger decrease in the distribution of motion artifacts after registration. It was found that the performance of DCE features as predictors of accuracy is sensitive to patient motion artifacts. We evaluated the performance of single DCE features as predictor with respect to registration. We find the AUC range shifted from 0.37 to 0.71 (with registration) to the range 0.44 to 0.64 (without registration). Pairwise analysis showed that the feature pair (*time-to-peak, initialAUC*) was not affected by the registration process.

### Study design

**Figure 1:**
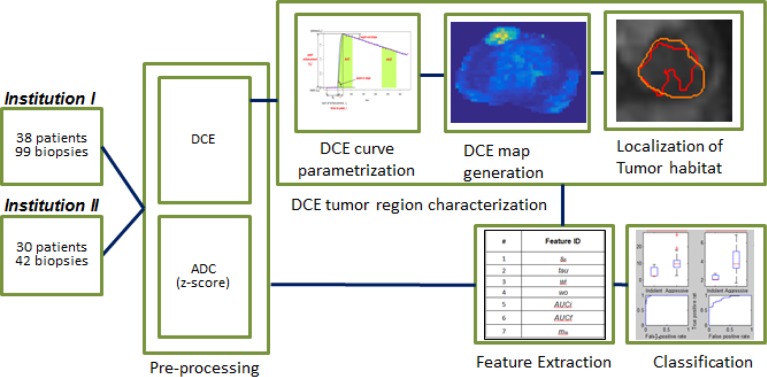
Block diagram of the overall processing A set of 38 patients from Institution I and 30 from Institution II with available mpMRI data were included in the analysis. Pre-processing included z-scoring of the ADC data and shifting/scaling of DCE data to the pre-contrast images. Voxel-wise parametrization of the DCE curves was performed and a DCE amp was generated for each parameter. A perfusion tumor habitat was localized from the DCE map based volume that was most similar to the radiology contour. Features from this DCE volume were computed for both DCE and ADC. A bottom-up approach to cluster important features was performed and a final model including 2 DCE and 2 ADC features is presented. Classification of these features was performed to evaluate prognostic value.

**Figure 2:**
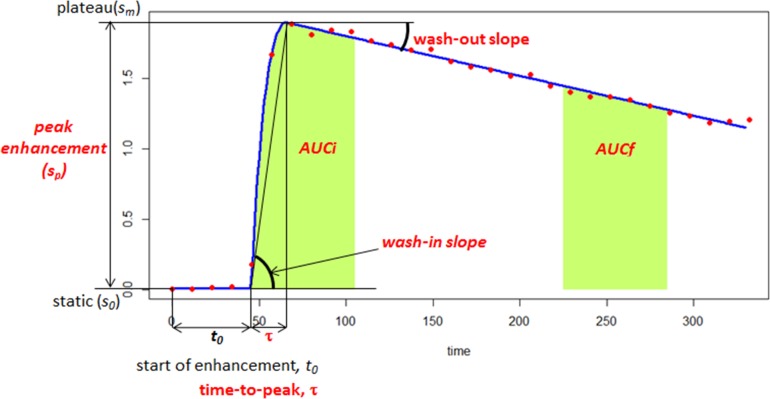
Quantitative modeling of the DCE-MRI time activity characteristics A 5-parameter curve is fitted to the DCE-MRI representative curve from the tumor habitat. The model consists of initial static intensity *s_0_*, plateau *s_m_*, start of enhancement *t_0_*, time-to-peak t, and wash-out slope *wo.* Peak enhancement *s_p_=s_m_-s_0_*; wash-in slope *w^i^*=*s_p_* / t. AUC_t1-t2_ is the area under the DCE curve (from red dots) between times t1 and t2. The AUFC_t1-t2_ is the area under the *fitted* curve (blue) between times t1 and t2.

**Figure 3:**
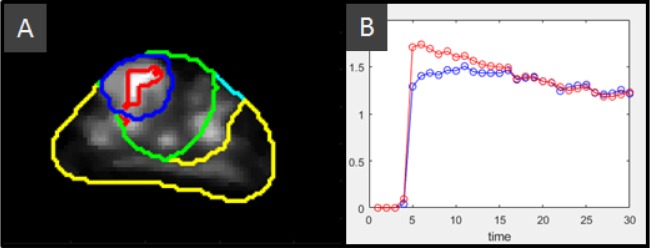
Definition of the *wash-in slope* habitat **(A)** Anatomical structures: Prostate (cyan), peripheral zone, PZ (yellow), and radiologist's lesion contour (blue) along with computed structures: A 3D 15 mm radius sphere (green) located at the center of mass of the marked lesion, and bounded by the prostate and the lesion's zone, in this case the transition zone. This bounded sphere is used as search space to select the region with large *wash-in slope*. The upper quartile is used to converge to the *wash-in slope* habitat (red). These structures are overlapped with the *wash-in slope* map that is computed by a pixel-wise fitting of the DCE time activity curves within the prostate. **(B)** Mean time-activity curves for the radiologist finding contour (blue) and the *wash-in slope* habitat (red). It can be seen that this habitat includes intra and peritumoral regions

The overall methodology of this study is shown in Figure 1. A *wash-in slope* map was generated by estimating the *wash-in slope* (Figure 2) of the time activity curves associated with the voxels within the prostate. The tumor region that was based on the radiologist finding on the T2W images was obtained. This region was centered based on the TRUS biopsy location that was imported directly from the fused TRUS/MRI system. This lesion boundary was initialized with a uniform 3D volume (extended region of fixed diameter) around the biopsy location and converged automatically into a *wash-in slope* habitat based on the upper quartile of the *wash-in slope* map (Figure 3A). The habitat's average absorption at each DCE time point was analyzed and used to generate time activity curves that were characterized by computing quantitative descriptors. These descriptors were then used in a classifier model to find features that discriminate clinically significant tumors. Intra-institution classification was used to select a set of DCE and ADC features that were analyzed in an inter-institution setting, where the model was build using the cohort from one institution and validated on the other institution. Inter-institution analysis of this subset of features was performed and the top performing features are shown in Table 5.

### Feature extraction

In this study, seven features were extracted from the DCE time activity curves, which describe both early and late enhancement (see Table 1). DCE curves were fitted using a bi-exponential model [26]. This semi-quantitative model has five parameters: initial static intensity *s_0_*, plateau *s_m_*, start of enhancement *t_0_*, time-to-peak *tau*, and wash-out slope, *wo.* Figure 2 shows an example DCE curve along with these parameters. Peak enhancement *s_p_=s_m_-s_0_*; wash-in slope *wi*=*s_p_* /*tau*. In addition, we computed two features that describe the area under the DCE curve between a time intervals, namely: AUC_t1-t2_ is the area under the bi-exponential fitted DCE curve between time, t1 and t2. *AUCi* = AUC_t0-t0+60_ measures the early wash-in uptake curve and *AUCf* = AUC_t0+240-t0+270_ measures the late wash-out curve. The seventh feature computes the multiplicative effect of wash-in and wash-out slopes and was computed as *m_io_*= wi^*^ wo. On the localized region, a set of 90 ADC features were computed consisting of intensity statistics, histogram and volume features. A subset of pairs of ADC features was obtained from the top performing pair-wise features selecting those with largest AUC.

### Computation of the *wash-in slope* habitat

The *wash-in slope* has been useful for cancer detection and localization [27], as well as in discriminating aggressive versus non-aggressive lesions [28]. It also differentiates prostate cancer from non-neoplastic lesions [29]. In [28] manual contours on whole mount histopathology after prostatectomy were mapped to T2 and the DCE wash-in slope was significantly different between these two groups for both the mean and the 75^th^ percentile within the mapped contour. In recent work [30], wash-in slope along with time-to-peak induced the highest sensitivity (0.89 for linear discriminant analysis, and 0.97 for SVM) for ovarian cancer. In this study, the *wash-in slope* parameter was used to converge to an intra and peritumoral region (*habitat*) around the biopsy location to characterize the surroundings of the biopsied lesion. This was done by first forming a sphere (radius *r* = 15 mm) around the given biopsy location to account for TRUS/MRI registration error. This region was bounded by the prostatic zone, either PZ or transition zone (TZ) allocating the largest lesion volume. The values for the wash-in slope within the localized sphere were used to obtain the region defined by the upper quartile. The corresponding DCE region will be our consensus tumor habitat region of interest. The mean DCE signal value at the consensus region at each sampling time was used as a representative perfusion curve for the patient biopsy. The definition of the *wash-in slope* habitat is shown in Figure 3

### Statistical analysis

Univariate analysis of the seven DCE features was performed to evaluate the overall discrimination of clinically significant to non-significant cancers using decision trees [31]. Sensitivity, specificity and AUC were computed on the features (see Table 1). Pair-wise multivariable analysis was performed by exhaustive comparison of all possible DCE feature pairs.

The underrepresented GS class was over-sampled using SMOTE [32], calibrated so that both classes had exactly the same size. Intra institution classifier performance was evaluated using *leave-one-out* (LOO) cross validation. For inter institution validation, a training model was built using the whole balanced data set in one institution, and tested using the unbalanced data set from the other institution. Data from different institutions were not mixed to build the classification models.

Pair-wise comparison of AUC was performed using DeLong test [33]. False discovery rate [34] (FDR) was used to correct for multiple comparisons.

Image processing and segmentations were performed on MIM Imaging PACS workstation (MIM Corporation, Cleveland, OH, USA). The feature computations were developed using custom code written in C++ and Matlab (Mathworks Inc., Natick, MA). Classifiers were implemented in Matlab. DeLong and FDR tests were performed in R.

## CONCLUSIONS

This paper describes a systematic approach to quantifying the clinical significance of lesions identified by radiology using a DCE-based habitat and evaluating both DCE and ADC features. Our approach identifies reproducible features for inter-institution prediction and can be translated seamlessly into clinical practice to guide radiologists and oncologists in the assessment of clinically significant prostate cancer.

## SUPPLEMENTARY MATERIALS TABLES






