# Erratum: Predicting clinically significant prostate cancer using DCE-MRI habitat descriptors

**DOI:** 10.18632/oncotarget.26802

**Published:** 2019-03-12

**Authors:** N. Andres Parra, Hong Lu, Qian Li, Radka Stoyanova, Alan Pollack, Sanoj Punnen, Jung Choi, Mahmoud Abdalah, Christopher Lopez, Kenneth Gage, Jong Y. Park, Yamoah Kosj, Julio M. Pow-Sang, Robert J. Gillies, Yoganand Balagurunathan

**Affiliations:** ^1^ Department of Cancer Physiology, H.L. Moffitt Cancer Center, Tampa, FL, USA; ^2^ Department of Radiology, Tianjin Medical University Cancer Institute and Hospital, Tianjin, China; ^3^ Department of Radiation Oncology, University of Miami Miller School of Medicine, Miami, FL, USA; ^4^ Department of Urology, University of Miami Miller School of Medicine, Miami, FL, USA; ^5^ Department of Radiology, H.L. Moffitt Cancer Center, Tampa, FL, USA; ^6^ Department of Cancer Epidemiology, H.L. Moffitt Cancer Center, Tampa, FL, USA; ^7^ Department of Radiation Oncology, H.L. Moffitt Cancer Center, Tampa, FL, USA; ^8^ Department of Urology, H.L. Moffitt Cancer Center, Tampa, FL, USA

**This article has been corrected:** During production, the author name for this article was given incorrectly. The proper author name is shown below.

**N. Andres Parra**

Original article: Oncotarget. 2018; 9:37125-37136. https://doi.org/10.18632/oncotarget.26437

