# Supplementary material for: Predicting clinically significant prostate cancer using DCE-MRI habitat descriptors
Source: Oncotarget. 2018 Dec 14;9(98):37125–36. doi: 10.18632/oncotarget.26437 (PMC6324677; doi:10.18632/oncotarget.26437)
Supplement: Supplementary file 1 [file oncotarget-09-37125-s001.pdf]

Predicting clinically significant prostate cancer using DCE-MRI habitat descriptors

SUPPLEMENTARY MATERIALS

Supplementary Table 1: Pairwise significance of difference between DCE feature tuples for Institution I. DeLong test was used with significance level set to 0.05. The 28 feature tuples correspond to those in Figure 2, flatten in row-major order.

See Supplementary File 1

Supplementary Table 2: Pairwise significance of difference between DCE feature tuples for Institution II. DeLong test was used with significance level set to 0.05. The 28 feature tuples correspond to those in Figure 2, flatten in row-major order.

See Supplementary File 2
