# Supplementary material for: Predicting clinically significant prostate cancer using DCE-MRI habitat descriptors
Source: Oncotarget. 2018 Dec 14;9(98):37125–36. doi: 10.18632/oncotarget.26437 (PMC6324677; doi:10.18632/oncotarget.26437)
Supplement: Supplementary file 2 [file oncotarget-09-37125-s002.docx]

**Supplemental Table 1:** Pairwise significance (p-value) to discriminate malignant to indolent using DCE feature tuples for **Institution I**. DeLong test was used with significance level (Type 1) set to α=0.05. The 28 feature tuples correspond to feature pairs as shown in *Figure 2*, flatten in row-major order
